# COPD Preferentially Disrupts Constitutive Redox Support Relative to Inducible Antioxidant Defense

**DOI:** 10.64898/2026.10.02.756280

**Published:** 2026-10-08

**Authors:** Alexis Hayes, Santiago Moreno-Caceres, Amber Delmas Eliason, David P. Marciano

## Abstract

Redox homeostasis requires both inducible antioxidant defense and constitutive machinery that continuously regenerates cellular reducing capacity. How these systems are altered across lung cell populations in chronic obstructive pulmonary disease (COPD) remains unclear. We integrated donor-level single-nucleus lung transcriptomes with smoke-exposure models, independent cohorts, and large-scale pharmacologic perturbation data. Both redox programs were reduced in established COPD, but constitutive redox support showed the greater loss, concentrated in fibroblasts and lymphatic endothelium. In fibroblasts, PGC-1α target-program output showed the most negative effect among 515 regulator programs despite preserved *PPARGC1A* expression. Smoke exposure activated inducible antioxidant defense across experimental models, whereas this adaptive program was reduced in established COPD. Pharmacologic perturbations further separated the two arms of redox homeostasis, with redox-directed interventions producing larger transcriptional responses in inducible defense and a distinct set of compounds increasing constitutive support together with PGC-1α target output and movement away from an independently defined COPD fibroblast state. These findings identify a cell-selective loss of constitutive redox support in COPD and implicate reduced PGC-1α target-program output in the fibroblast redox phenotype.

## Full Text Availability

The license terms selected by the author(s) for this preprint version do not permit archiving in PMC. The full text is available from the preprint server.

